# Design of Tunable Holographic Liquid Crystalline Diffraction Gratings

**DOI:** 10.3390/s20236789

**Published:** 2020-11-27

**Authors:** Katarzyna A. Rutkowska, Anna Kozanecka-Szmigiel

**Affiliations:** Faculty of Physics, Warsaw University of Technology, 00-662 Warszawa, Poland; anna.szmigiel@pw.edu.pl

**Keywords:** holographic diffraction gratings, photoalignment, liquid crystals

## Abstract

Tunable diffraction gratings and phase filters are important functional devices in optical communication and sensing systems. Polarization gratings, in particular, capable of redirecting an incident light beam completely into the first diffraction orders may be successfully fabricated in liquid crystalline cells assembled from substrates coated with uniform transparent electrodes and orienting layers that force a specific molecular distribution. In this work, the diffraction properties of liquid crystal (LC) cells characterized by a continually rotating *cycloidal* director pattern at the cell substrates and in the bulk, are studied theoretically by solving a relevant set of the Euler-Lagrange equations. The electric tunability of the gratings is analyzed by estimating the changes in liquid crystalline molecular distribution and thus in effective birefringence, as a function of external voltage. To the best of our knowledge, such detailed numerical calculations have not been presented so far for liquid crystal polarization gratings showing a *cycloidal* director pattern. Our theoretical predictions may be easily achieved in experimental conditions when exploiting, for example, photo-orienting material, to induce a permanent LC alignment with high spatial resolution. The proposed design may be for example, used as a tunable passband filter with adjustable bandwidths, thus allowing for potential applications in optical spectroscopy, optical communication networks, remote sensing and beyond.

## 1. Introduction

Diffraction gratings are important optical components used in the field of spectroscopy by chemists, biologists and physicists [1,2,3]. High-quality elements of this type require precise control over periodic surface modulation at the subwavelength scale and thus advanced technologies such as optical beam lithography, scanning beam holography, direct electron beam lithography or photo-lithography are involved in their fabrication. When it comes to optical communication and information processing, gratings with controllable diffraction properties may serve as essential functional elements allowing for effective beam switching, steering, filtering, shaping, splitting or combining. For these specific applicational purposes, tunable periodic structures, dynamically adjusted with the use of external fields and factors [4,5,6,7,8] and additionally generating light beams at relatively large deflection angles are of particular significance [9,10,11]. Additionally, from a practical point of view, when using diffraction gratings to redirect light beams and to change their paths in space, it is beneficial to eliminate the zeroth-order beam completely. Various techniques, such as introduction of a third phase level to the original binary grating profile, inclusion of diffractive micro-prisms or Fresnel lens in the design, introduction of mechanical mask to block the optical signal at an intermediate image plane, application of double-sided diffraction elements or application of holographic gratings [12] may be used to complete this task. In principle, such proposed solutions allow for the signal in the zeroth diffraction order to be lowered to the null level, with the diffraction orders higher than the first one, also present in most cases.

Liquid crystals (LCs) with their peculiar optical properties (including significant birefringence and extended spectral transparency) have been successfully demonstrated as active media for switchable grating fabrication [8,13,14]. In many approaches, the grating structures of this type are obtained by generating a periodically varying director orientation in the bulk of nematic LC (NLC) cell, while their tunability is enabled by applying electric voltage across the structures [15,16,17,18]. Among various director alignment configurations, the ones showing either a rectangular or so-called *cycloidal* [19,20] (i.e., monotonously rotating by 180° along a grating vector) pattern are the most attractive for practical realization of beam splitting or steering devices [21,22,23,24,25,26]. The corresponding binary and polarization gratings enable, theoretically, almost complete or fully complete switching between the zeroth and first diffraction orders [12,27,28]. The fabrication process of such gratings is often based on a photoalignment technique that involves either photomasking or holographic exposures of empty cells assembled from the glass substrates coated photosensitive orienting layers. It is contrary to other possible methods for periodical structures to be created in liquid crystalline layer, when complex micro-structuring of the driving electrodes is required [29].

When designing polarization gratings with a *cycloidal* director pattern, an important issue is to choose properly a cell gap in respect to a grating spacing so that the periodical molecular arrangement of a specific type may be preserved in the LC layer thickness [30]. Further, the cell gap and LC birefringence determine the phase difference between ordinary and extraordinary waves behind the structure, thus affecting diffraction the properties of the grating. What is more, a significant electro-optic response of LC allows for the creation of flexible and voltage-tunable architectures when transparent conductive coatings are sputtered on glass substrates. Accordingly, by matching the material and geometric parameters of the grating, as well as applying an electric field of a certain level, the wavelength(s) may be achieved, fully diffracted into the first order(s) [12,30]. Keeping in mind that synthesizing new LC materials and mixtures offers a possibility to modify their birefringence, electric anisotropy (together with its sign), viscosity, elastic constants, phase transition temperature and optimization of device characteristics are relatively easy to achieve. Therefore, several important parameters of LC-based gratings of this type, such as threshold voltage, maximum steering voltage, switching times, spectral properties or thermal operation range may be tuned quite effectively.

In this work, we theoretically investigate the diffraction properties and electro-optical characteristics of LC polarization gratings formed by using typical NLCs. While the response times are determined not only by material parameters but, more importantly, depend quadratically on the LC layer thickness, the cells with a 5 or 3 µm-gap (typically used in LC displays) are considered capable of reasonably fast switching (in the orders of tens of milliseconds). The gratings with relatively low periods, that is, in a range of 5 ÷ 20 µm, are studied, so that the diffracted beams are generated at comparatively large angles. By analyzing analytically the proposed configuration and by solving numerically differential (Euler-Lagrange) equations, we demonstrate how the first order diffraction efficiency spectra are affected by the cell gap and LC birefringence at zero voltage, as well as how it changes when the LC cell is electrically biased. Moreover, we predict the values of tuning voltages that ensure the achromaticity of gratings in the spectral range 370 ÷ 900 nm. The results of numerical calculations are fully consistent with the preliminary estimates and experimental demonstrations presented in our previous works [31,32]. Possible applications might then be anticipated in the photonic systems, sufficient for moderate response times.

## 2. Materials and Methods

**Figure 1:**
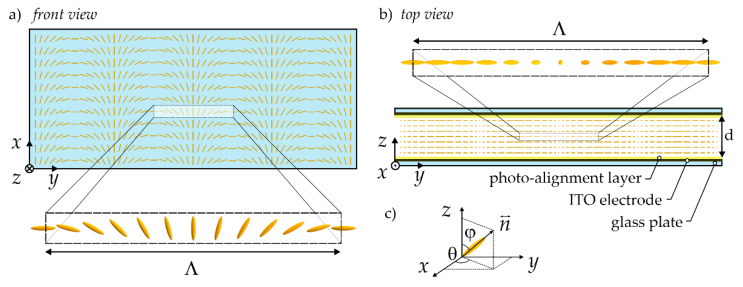
Scheme of the liquid crystalline diffraction grating under consideration. (**a**) Front view of the liquid crystal (LC) cell with a periodic molecular orientation on the glass plate; (**b**) Top view of the LC cell with its main elements indicated. The thickness of the cell (i.e., the cell gap)—*d*, the period of the *cycloidal* distribution of LC molecules—Λ. Please note that the light beam propagates along the *z*-axis when illuminating the grating. (**c**) Definition of the LC molecular arrangement in space with the use of director $\overset{↔}{n}$ and two orientation angles: φ and θ. Note that the illustrations are not drawn in scale.

Our studies assume that the tunable holographic diffraction grating is formed in a typical LC cell, consisting of glass plates uniformly covered with conductive Indium Tin Oxide (ITO) coatings and with alignment layers forcing a specific molecular orientation within the liquid crystal bulk (see Figure 1b). The considered grating geometry implies an ideal *cycloidal* distribution with the period of Λ along the *y*-axis. It means that the spatial distribution of the director, that is, the directionless unit vector defining the orientation of the LC molecules, is described by the cos(π*y*/Λ) function on the glass surfaces limiting the cell (as shown in Figure 1a). In the absence of disturbances and external fields, it is deemed that such a periodic distribution of the director forced by the boundary conditions is perfectly reproduced along the thickness of the liquid crystal layer.

It is worth noting that the imposed periodical conditions for the arrangement of liquid crystal molecules are accessible by means of the photo-alignment process, in which an empty cell coated with photosensitive layers is exposed to two coherent beams with opposite circular polarizations crossing at a small angle [24,30,31]. Exemplarily, the photo-orienting material reported in our previous works [31,32] enables permanent recording of liquid crystalline diffraction gratings with periods of 20 or even 10 micrometers, whilst providing a perfect, high-resolution reproduction of the interference pattern that is required in holographic registration of the best-quality *cycloidal* structures. With a rotating director accurately recorded, it is possible to successfully exploit such a property of analyzed gratings so that there are no higher orders of diffraction present except the first one and furthermore, it is possible to obtain output light beams directed in a specific spatial direction (i.e., corresponding only to +1 or only to −1 diffraction order [12,24,30]) depending on the polarization state of the input light beam. In addition, it has been demonstrated that polarization gratings recorded in proper media by means of a polarization holography technique, in particular with the use of two coherent beams of opposite circular polarizations, possess unique diffraction properties and that a first-order diffraction efficiency up to 100% may be achieved even in thin gratings (operating in the Raman-Nath regime) [28]. Such a theoretical prediction has been confirmed experimentally by References [24,25,31].

There are analyzed grating structures with different periods (Λ) equal to 20 µm, 15 µm, 10 µm, 7 µm or 5µm and thicknesses (*d*) of either 3 or 5 µm. As already mentioned, selected spacings Λ are easily obtained in practice with a polarization holography technique by changing the crossing angle between the two coherent beams or/and the wavelength of a laser source used in the photoalignment process. Specifically, for a 442-nm recording wavelength and exposure angles not larger than 10 degrees it can be roughly estimated that the minimum period of the polarization pattern is about 2.5 µm (still not reported for holographic polarization gratings based on NLCs). The period as small as 4 µm has been obtained experimentally by Sarkissian et al. [30] and similar value should be reachable by photo-patterning of azobenzene containing aligning layers.

The chosen grating thicknesses, being in practice the LC cell gaps, are also easily available with accessible glass spacers used when assembling the cells. In order to meet the condition formulated by Sarkissian et al. [30] for transferring the *cycloidal* director profile prescribed at the cell surfaces into the bulk of the cell, the LC grating structures with periods of 20 µm, 15 µm and 10 µm are considered when performing simulations for the cells with gaps of 5µm, while smaller grating periods of 10 µm, 7 µm and 5µm are assumed for the 3 µm-cells.

The realization of a tunable diffractive optical element in the proposed system is possible owing to an electro-optic response of LC materials, behaving as birefringent uniaxial with their optical axis coinciding with the molecular director. It permits to transform the LC layer with a specific periodic molecular arrangement and thus a required refractive index spatial distribution, into a diffraction grating of specific characteristics. In the ideal case of the planar configuration considered here, the long axis of LC molecules is perfectly aligned in the *x*-*y* plane (with a *cycloidal* distribution along the *y*-axis imposed by the boundary conditions at the cell interfaces) when no voltage is applied. In an NLC with positive low-frequency electric anisotropy (as is for all analyzed materials, see Table 1), the molecules tend to orient along the direction of an external electric field (here the *z*-axis). In the present case, the reorientation process is of a threshold nature (governed by the Fredericks condition), allowing for the change in orientation angles and thus in optical properties of an LC layer and diffraction characteristic of the grating itself. Both the initial and electrically-driven molecular arrangement, specifying an effective refractive index distribution within LC layer, can be efficiently modeled, based on the continuum theory for NLCs [33].

The relevant computer simulations have been performed with the use of self-derived differential equations and solved using numerical schemes based on the successive-over-relaxation (SOR) method [34]. For this purpose, the total free energy with its term depending on electric field together with terms describing the elastic contributions (as expressed through Frank’s formalism) has been taken into account. By assuming that the LC arrangement (with the orientation of the molecular long axes defined by the director) is described by the pair of the polar angles (φ, θ) determining the tilts from the *z*-axis and in the *x*-*y* plane respectively (see Figure 1c), the free energy density of the system has been minimized. It has resulted in the differential (Euler-Lagrange) equations governing, in general, the relation between the orientation angles and the applied voltage. The low-frequency electric field spatial distribution, to be included in such deliberations, has been calculated from Maxwell’s relations in the presence of the specific electric potential. It is worth underlining that the spatial distribution of the electric field related to the local reorientation of the electric tensor must be considered. Specifically, the full form of the electric permittivity tensor has been included in our numerical simulations, with its nine elements given by the relation: ε*_ij_* = ε_⟂_δ*_ij_* + Δε *n_i_n_j_* (where ε_⟂_ is the electric permittivity in the direction perpendicular to the director, δ*_ij_* is the Kronecker delta, Δε is the electric anisotropy and *n_i_* is the *i*-th component of the director). In the analyzed case, $\overset{↔}{n}$ = [sinφcosθ, sinφsinθ, cosφ], which means that the electric permittivity tensor depends on the orientation angles φ and θ. At the same time, the relation ∂/∂y(ε*_yy_* ∂V/∂y) + ∂/∂z(ε*_zz_* ∂V/∂z) = 0 is used to calculate electric potential V in the *y-z* plane for a given spatial distribution of orientation angles. This makes our numerical calculations for the orientation angles (with the use of the Euler-Lagrange equations) and then for the electric potential, repeated in the successive iterations—both with the use of relaxations schemes—so many times as further changes in calculated values are not observed at an assumed level of convergence tolerance. In contrast to our previous works [31,32], the full form of the Euler-Lagrange equilibrium equations has been taken into account, without special conditions imposed on the elastic constants (the most general case has been considered with K_11_ ≠ K_22_ ≠ K_33_). By assuming strong anchoring as the boundary conditions for the director alignment at a liquid-crystal-solid interface, which is fully reasonable for the intended photo-alignment treatment of cell surfaces, the spatial distribution of director may be derived for any uniaxial nematic and for any value of the electric field applied to the cell of a given gap. With the orientation angles (φ, θ) determined in this way, the effective refractive indices for the *x*- and *y*-polarized waves (and thus effective birefringence, Δ*n*_eff_ =|*n*_eff,*y*_(φ, θ)—*n*_eff,*x*_(φ, θ)|) can be calculated as a function of ordinary (*n*_o_) and extraordinary (*n*_e_) refractive indices of the LC material under consideration.

**Figure 2:**
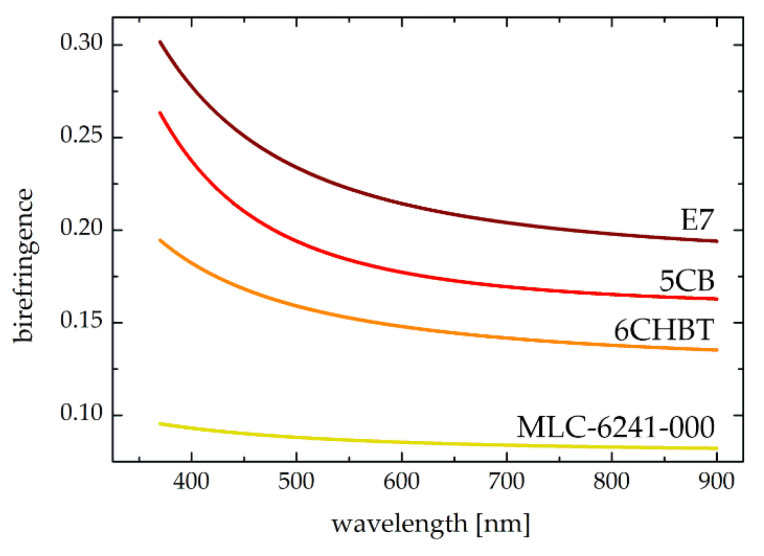
Birefringence dispersion for typical nematic liquid crystals and mixtures [35,38,40] assumed in numerical simulations concerning the design of holographic diffraction gratings.

The calculations have been made in accordance with real parameters of well-known liquid crystalline materials, extensively described in the literature, such as: 6CHBT (synthesized at the Military University of Technology in Warsaw, as analogue to 6CPS [35]) [36], E7 [37,38] and 5CB (also known as PCB) [38,39,40], the latter two commercially available from Merck. Additionally, in view of the feasibility of shortening switching times and other application aspects [41], the possibility of creating diffraction gratings with the use of low-birefringent liquid crystals has also been investigated. For this purpose, the Merck mixture typically used in TFT display technology, namely MLC-6241-000 [38,40,42] has been analyzed. Table 1 contains selected physical parameters of the liquid crystals under consideration, including elastic constants [36,37,39,42], electric permittivity at low frequencies [36,37,42] and ordinary and extraordinary refractive indices for the sodium D-lines at room temperature [35,38,40], while Figure 2 shows the birefringence (Δ *n*= *n*_e_ − *n*_o_) dispersion of these liquid crystalline materials.

Large electric permittivity anisotropy (~10 for three liquid crystalline materials listed in Table 1) guarantees significant reorientation in LC molecular arrangement (leading in turns to large modifications of optical properties) for relatively weak electric fields (in a range of 1 ÷ 2V/μm). In considered geometry, the Fredericks thresholds for the electric field (*E*_th_) [43] are estimated to be 0.83, 0.82, 0.74 and 1.47 V/μm for 6CHBT, E7, 5CB and MLC-6241-000 LC, respectively.

## 3. Results and Discussion

Numerical calculations have been performed in such a way that firstly the spatial distribution of the director has been determined and, on this basis, the effective refractive indices (for *x*- and *y*-polarized light) of the liquid crystalline layer have been derived. As already mentioned, the latter results from the arrangement and positioning of the long axis of LC molecules in the sample volume, giving rise to effective birefringence (Δ*n*_eff_) when integrated through the cell thickness and subtracted from each other. After reaching the value of Δ*n*_eff_ for all wavelengths, it is possible to determine the spectral characteristics of diffraction efficiency for the LC cell of specific geometry, in particular, the first order diffraction efficiency (DE_1_) of the *cycloidal* diffraction grating with the period of Λ, thickness of *d* and for the incident light with the vacuum wavelength of λ and linear/circular polarization equal to sin^2^(π*d*Δ*n*_eff_λ^−1^) [24]. The latter applies in the ideal case with all losses (related, e.g., to scattering and reflections) omitted. That means that 100% DE_1_ is theoretically possible when the retardance (i.e., difference in the phase shift between two orthogonal polarizations, 2π*d*Δ*n*_eff_λ^−1^) is an odd multiple of π. It corresponds to the multiples of the half-wave conditions for *d*Δ*n*_eff_, taking the values of (*m* − 1/2)λ, with *m* integer and Δ*n*_eff_ being the function of the wavelength (λ) and the applied voltage (V). It is worth noting that the total losses at the level of about 20–30% may be estimated on the basis of our previous experimental works [31,32].

**Figure 3:**
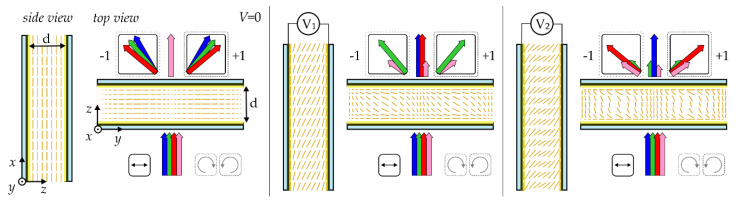
Schematic representation of the diffraction characteristics of exemplified liquid crystalline gratings with the *cycloidal* director pattern at the cell substrates. Please note that, in general, only the 0 and/or ±1 diffraction orders are present at the output of the LC cell, regardless of input polarization. The wavelengths registered in the specific spots (i.e., diffraction maxima) depend on the sample retardance, specifically with *d*Δ*n*_eff_ = *m*λ for 100% diffraction efficiency in the zeroth—(DE_0_) and with *d*Δ*n*_eff_ = (*m* − 1/2)λ for 100% diffraction efficiency in the first- (DE_1_ ≡ DE_+1_ + DE_−1_) diffraction order. The effective birefringence of a liquid crystalline material (Δ*n*_eff_) depends on the wavelength (represented schematically by the arrows of the different colors) and applied voltage. A circularly-polarized beam is diffracted into +1 or −1 order, depending on its handedness, whereas both first orders (±1) are present for a linearly-polarized input beam with their efficiencies (DE_+1_ and DE_–1_) giving together 100% in the most favorable condition. Shorter arrows at the output of the cell represent the wavelengths which are out of the retardation conditions mentioned above.

The top and side views of LC cells with a detailed representation of molecular orientation (retrieved from numerical simulations) for no voltage and for two different voltages (*V*_2_ > *V*_1_) are exemplarily shown in Figure 3. When the applied voltage exceeds the threshold value, the reorientation within the LC layer takes place, varying the effective birefringence of the system, whereby light at different wavelengths is coupled into the zeroth–and the first-order(s), respectively. Please note that the cases for some random voltages are presented in Figure 3. In principle, it is possible to adapt geometrical parameters of the cell (*d* and less importantly Λ) together with material parameters of LC material and/or a value of applied voltage to obtain the grating deflecting particular wavelengths and moreover eliminating the zeroth-order beam from a specific spectral range completely. What is additionally important for the holographic gratings of the *cycloidal* type is the fact that zeroing of diffraction efficiency for the zeroth order guarantees that a linearly polarized light beam after passing the grating is redirected only in the first diffraction orders (i.e., ±1) or only in one specific direction (i.e., +1 or −1 diffraction order) when a circularly-polarized light beam is used at the input (see the schematic interpretation in Figure 3). Such a way of operating was experimentally demonstrated in Reference [31] for an analogical system. Furthermore, the validity of our theoretical model presented here may be confirmed by the experimental data presented in our previous works [31,32] for the LC cells with thicknesses of about 5 µm, infiltrated with 5BC LC and with a grating period of 20 µm and 10 µm, respectively.

**Figure 4:**
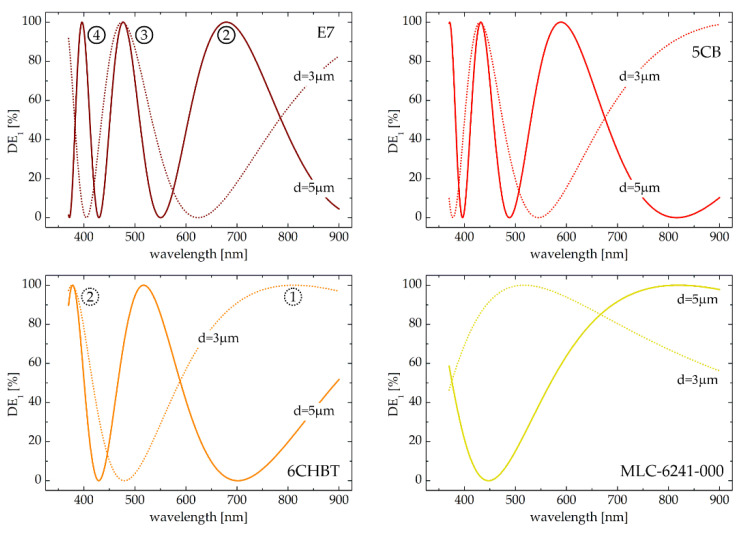
Spectral dependences of the first order diffraction efficiency (DE_1_) for selected liquid crystalline materials and for two different thicknesses of LC cell (*d* = 5 µm—solid lines; *d* = 3 µm—dotted lines) with no voltage applied. For the fixed cell gaps and specific LC material the obtained result does not depend on the grating period (Λ = 20 µm, 15 µm, 10 µm for *d* = 5 µm and Λ = 10 µm, 7 µm, 5 µm for *d* = 3 µm). Maxima are numbered sequentially starting from the longest to the shortest wavelengths, as determined by the condition for the phase retardance.

In Figure 4 the first order diffraction efficiency spectra at zero voltage are presented for four selected liquid crystalline materials and for two different thicknesses of LC cell. Note that the minima in such spectral characteristics correspond to the wavelengths that are directed into the zeroth order entirely, while the maxima stand for the wavelengths that are completely redirected to the first diffraction order. In general, the number of possible maxima depends on the values taken by normalized retardation (*d*Δ*n*_eff_λ^−1^) within the analyzed spectral range. The first maximum (for *m* = 1) appears for the wavelength at which the effective birefringence is equal to the wavelength divided by the doubled cell thickness. Subsequent maxima (if observed within a given spectral range) require higher values of normalized retardation, which is equal to 3/2 and 5/2 for the second (*m* = 2) and for the third (*m* = 3) maximum, respectively. Forthcoming spectral peaks are shifted towards the shorter wavelengths and their bandwidths (inversely proportional to Δ*n*_eff_) are narrowing as shown in Figure 4 and as indicated by exemplified numbering of the peaks in the left panels. Please note that the characteristics obtained for a particular LC and the cell gap do not change when varying the grating period. This is because the effective birefringence is equal to the material birefringence itself (Δ*n*_eff_ ≡ Δ*n*) when no voltage is applied to the sample. However, even if spectral dependences of the first order diffraction efficiencies for no electrically biased grating are not directly dependent on the grating period, one has to keep in mind that this parameter is particularly important for practical implementation as a beam-steering device. Keeping the period suitably small, a substantial separation angle between the propagation directions of the zeroth- and first-order beams may be obtained. On the other hand, the structure period has to be adapted to the gap between the glass plates so that the particular *cycloidal* pattern is preserved in the bulk of the cell. The fact that these two geometrical parameters cannot be changed separately from each other, with their ratio maintained at the specific level [30], makes the grating considerably thin when small period is required. On the one hand, this is positive, because a narrower cell gap leads to faster response times but on the other hand, it requires a higher birefringence to satisfy the minimum value of *d*Δ*n*_eff_ requirement (i.e., equal to λ/2) within the spectral range under consideration.

When examining the graphs shown in Figure 4 and searching for the condition in which grating redirects almost all optical signal into the first diffraction order, one can conclude that for a thicker cell (i.e., for *d* = 5 µm) and a low-birefringent LC (MLC-6241-000) the operation spectral range is relatively broad with DE_1_ ≥ 95% from 725 nm to 900 nm (and above). By increasing the liquid crystalline birefringence, the grating which does not diffract an optical signal from near-infrared (NIR) is obtained. At the same time, multiple spectral fringes over the visible are observed and such a device effectively directs the light of selected wavelengths completely into the ±1 diffraction order, which is: green and violet (6CHBT); red, blue and violet (5CB), red, green and blue (E7). After reducing the cell thickness to 3µm, the number of spectral maxima observed for the first order diffraction efficiency for 5CB, 6CHBT and E7 in the visible is decreased, however a broadband maximum appears in the infrared. For low-birefringent grating (i.e., for MLC-6241-000) the operation spectral range (with DE_1_ ≥ 95%) is shifted from the NIR to visible, taking the values from 460 nm to 590 nm. It allows to state that a change in the cell thickness (from 5 to 3 µm) significantly modifies the spectral characteristics for DE_1_ while blue-shifting the wavelength at which the grating diffracts optical signal entirely into the first order by 300 nm. The impact of the cell gap is also noticeable for highly—(E7) and moderately-birefringent (5CB, 6CHBT) liquid crystals when comparing the solid and dotted lines in each graph in Figure 3. In fact, the difference in the birefringence of these three particular materials influences the obtained characteristics much less than the change in the cell thickness does. Nevertheless, by modifying the liquid crystalline material infiltrating the cell whereas keeping the geometrical parameters of the sample constant, one may adjust the grating characteristics and maximize its first order diffraction efficiency in a totally different spectral range. With no electric field applied and for an LC cell with a thickness of 5 µm, the most extreme maxima, when it comes to the longest possible wavelengths (in all cases *m* = 2), are obtained at the wavelengths of 680 nm, 590 nm and 519 nm for E7, 5CB and 6CHBT LC, respectively. Defining the satisfactory level of the first order diffraction efficiency at 95%, one can see that the latter occurs for the spectral range (Δλ_95%_) of 50 nm, 40 nm and 35 nm, respectively. In addition, further maxima for *m* = 3 and possibly for *m* = 4 (E7, 5CB) are observed at short wavelengths with the peaks of significantly narrow widths (Δλ_95%_ ≤ 10 nm). As already mentioned, when a low-birefringent material is used instead, in the same geometrical configuration, the wavelength, at which the first order diffraction efficiency reaches the value of 100%, is represented by the single maximum (*m* = 1) in the NIR with Δλ_95%_ of almost 200 nm.

It seems that by changing the birefringence of a liquid crystalline material (possibly taking also one out of the list proposed here) and by varying the cell thickness, one can find optimal conditions to meet predefined requirements and get the grating to diffract the signal from the specific spectral range completely into the first order (with the zeroth order beam totally vanished). However, from the practical point of view, even more important is the ability to dynamically adjust the diffraction characteristic of the grating and thus tune the output signal. From the results of the numerical simulations, as well as from our previous experimental experiences, it can be found that the application of a low-frequency electric signal with the amplitude of a single volt is high enough to significantly modify the holographic liquid crystalline grating specification and its operation. Illustrative diagrams showing the change in the molecular orientation due to the applied electric field are presented in Figure 3. Such transformation in molecular arrangement influences the effective refractive indices and thus the effective birefringence Δ*n*_eff_ of LC layer, too For this reason, it is possible to dynamically change the value of normalized retardation (*d*Δ*n*_eff_λ^−1^), which in turns determines the diffraction efficiency of the analyzed grating. In general, when an electric field above the threshold value is applied, the maxima of the first order diffraction efficiency are shifted towards the shorter wavelengths (i.e., the effect of applied voltage on the spectrum is a blue-shift). It is so due to the nematic director out-of-plane reorientation caused by external voltage, which decreases the effective birefringence (with Δ*n*_eff_ = Δ*n*_eff_(V)). Importantly, as already mentioned, the voltage threshold of initiate electrical tuning is less than 1 ÷ 1.5V for the analyzed LC materials.

**Figure 5:**
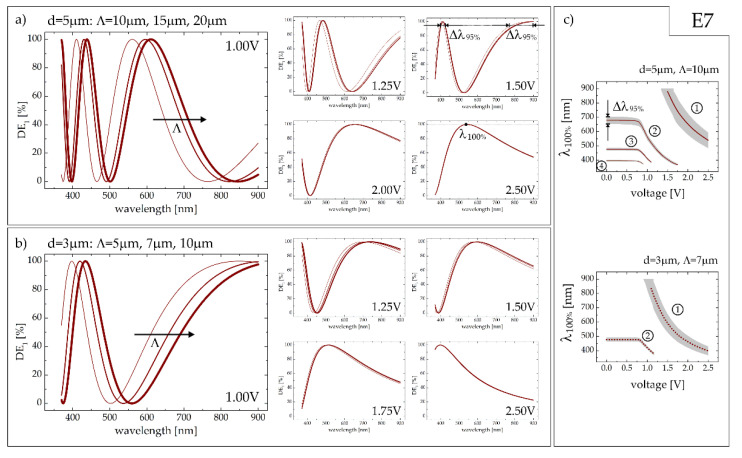
Spectral dependences of the first order diffraction efficiency for E7 LC in the electrically biased cell of a thickness of (**a**) 5 µm and (**b**) 3 µm. the value of applied voltage is given in each graph. Thicker lines in panels (**a**,**b**) indicate grater periods of the diffraction grating. (**c**) The wavelength at which the maxima of the first order diffraction efficiency are observed (solid lines for *d* = 5 µm and dotted lines for *d* = 3 µm) with the gray shadows indicating the spectral ranges (Δλ_95%_) in which DE_1_ ≥ 95%.

Results presenting electric tunabilty of proposed device on the example of the grating infiltrated with E7 LC and with a thicknesses of 5 and 3 μm are shown in Figure 5. As in contrast to the case with no voltage applied, the modification of the grating period when keeping the LC cell thickness unchanged affects the first order efficiency spectra with their maxima slightly shifted towards the shorter wavelengths with a decreasing grating period. However, the higher the voltage, the more period-independent results are.

The thicker cell (*d* = 5 μm), providing initially (i.e., with no electric bias) the maxima of DE_1_ for the wavelengths of about 400 nm, 475 nm and 680 nm (see Figure 4), can be effectively and dynamically tuned within the visible (VIS) spectral range with the voltages as low as 0.9 ÷ 1.5V (as shown in the top graph in Figure 5c). Please note, for instance, that at a wavelength of 500 nm the minimum of DE_1_ for V = 1.00 V is changed into the maximum when the applied voltage is equal to 1.25 V. Moreover, the peak observed at 680 nm with no voltage (Figure 4) is shifted to 405 nm with 1.50 V (Figure 5a). The spectral shift of this specific peak may be tracked more accurately when reading the solid curve for *m* = 2 in Figure 5c. It is worth underlining that it is possible to make the same grating functional within Δλ_95%_ = 590 ÷ 735 nm when a higher voltage of about 2.0 V is applied (Figure 5a). In fact, the NIR/red tuning (i.e., in 700 ÷ 900 nm spectral range) of the maximal first diffraction efficiency is possible for voltages of 1.5–2.0 V (for the peak corresponding to *m* = 1 when analyzing the retardation condition, see Figure 5c). When increasing the voltage even more, the single maximum (blue-shifted with the electric bias) is observed within the spectral range under consideration (λ_100%_ = 540 nm, Δλ_95%_ = 108 nm for V = 2.5 V, see Figure 5a). A further rise in applied voltage does not lead to significant changes in diffraction characteristics of the grating. The saturation of reorientation process forces molecular orientation to be flatten along the *z*-direction, thus lowering the effective birefringence. The latter may be completely diminished when homeotropic alignment is achieved but such configuration is beyond the scope of this work.

By decreasing the cell thickness to *d* = 3 µm, the reduction of phase retardation values and thus decrease in the number of DE_1_ maxima accessible within the analyzed spectral range are observed. Voltage changes from 1.0 V to 2.5 V allow for complete achromaticity of the grating in the full IR-VIS spectral range to be achieved. Notably, the relatively broad peak (with Δλ_95%_ from 100 to 200 nm), observed firstly at 900 nm for 1.00 V (Figure 5b), is smoothly blue-shifted throughout the analyzed spectrum down to the wavelength of 400 nm for V = 2.50V. Additionally, the thinner the cell, the smaller the grating period may be considered. It is particularly important from the application point of view while allowing for significant spatial separation between zeroth-order and deflected beams. In particular, for the geometrical parameters of the liquid crystalline gratings considered here (with the assumed minimal grating period achievable with the use of available photo-alignment technology) one may estimate the deflection angles of about 15° (Λ = 5 μm) for a probe wavelength of 633 nm, which is in the middle of the considered spectral range. However, although such analytical predictions give some physical insight and enable reasonable work on some aspects, additional phenomena and restrictions must be included, limiting accessible deflection angle [20].

Short-wavelength maxima appearing for the electric fields below the Fredericks threshold (*m* = 3 and 4 for *d* = 5 µm; *m* = 2 for *d* = 3 µm), even if useful for monochromatic applications, are barely tuned with voltage, almost instantly shifted beyond the interesting spectral range.

**Figure 6:**
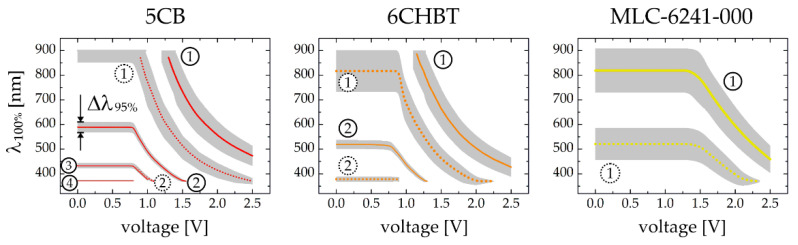
The wavelengths corresponding to the maxima of the first order diffraction efficiency (DE_1_) as the functions of applied voltage. The lines represent the spectral positions of the maxima (λ_100%_) while the gray shadows behind them illustrates the spectral ranges for which DE_1_ ≥ 95% (Δλ_95%_). Results are numerically calculated for selected liquid crystalline materials (5CB, 6CHBT, E7) and for two different thicknesses of LC cell (*d* = 5 µm, Λ = 10 µm—solid lines; *d* = 3 µm, Λ = 7 µm—dotted lines). Numbering of successive maxima, defined as the multiple of the half-wave condition for *d*Δ*n*_eff_, is indicated in each case.

Comprehensive graphs summarizing the dependence of the wavelength corresponding to 100% of the first order diffraction efficiency and the spectral range for DE_1_ ≥ 95% (both parameters, λ_100%_ and Δλ_95%_ introduced and marked earlier in Figure 5 for clarity) on the applied voltage for different liquid crystalline materials and for different cell gaps is shown in Figure 6. Such diagrams, obtained thanks to detailed numerical simulations performed, provide full information about optimum correlation between the grating period, the cell thickness and the liquid crystalline birefringence to be satisfied in order to maximize the diffraction efficiency tunablility for applied voltages and accessible spectral range. In fact, they may act as informational charts to support the design process of diffraction grating of required characteristics and particular applicational purposes.

## 4. Conclusions

In this work theoretical considerations have been presented (supported with advanced numerical simulations) on the relations between details in the design of periodically aligned liquid crystals and the performance of such structures as efficient diffraction gratings. Attention has been particularly focused on the LC cells with uniform electrodes and aligning layers, imposing the so-called *cycloidal* director pattern, which is well-known for the capability to diffract an incident beam into the first orders alone. In the studies, four typical nematic substances and mixtures, two different cell gaps (i.e., 3 and 5 μm) and the spacing of the director pattern lying in a range of 5 ÷ 20 μm, have been specifically considered. By solving the differential (Euler-Lagrange) equations numerically, the first order diffraction efficiency spectra of each analyzed grating have been determined in the spectral region of 370 ÷ 900 nm. The effects of liquid crystal birefringence and cell gap on the position and number of curve maxima (corresponding to the wavelengths that are completely diffracted into the first order) have been demonstrated and discussed. Specifically, the changes in liquid crystal molecular distribution (and thus in effective birefringence) arising from the electric voltage applied across the cells have been calculated for the voltages from the threshold value up to 2.5 V. The obtained results demonstrate the effect of the blue-shift of diffraction efficiency curves and the possibility of continuous tuning of grating operational wavelengths to any desired one(s) from the analyzed spectral range. In particular, for the cells of 5-μm-thickness filled with E7, 5CB or 6CHBT (which represent high- and moderate-birefringent LCs), it has been shown that the voltages as low as about 1.5 V are enough to shift the operation wavelengths from these lying in the red or green - to the violet region, while slightly higher voltages (reaching the value of about 2 V) allow for shifting the DE_1_ curve maxima from near IR to red wavelengths. By employing the LC cells of smaller thickness, that is, of 3 μm, the steering voltages needed for adjusting the curve maximum in the latter spectral region are even lower. In the case of the cell filled with a low-birefringent liquid crystal, a thicker cell guarantees grating tunability in the whole studied spectral region, that is, 370 ÷ 900 nm. The application of the 3-μm-thick cell in the case of a liquid crystalline material with birefringence less than 0.1 (MLC-6241-000 herein) does not allow for tuning in the NIR/red spectral range.

It has to be once again underlined that the proposed configuration provides considerable potential for engineering the diffractive properties of the grating by dynamically adjusting its parameters. In fact, it represents an excellent example of the system with tunable characteristics, where the grating period and the effective birefringence can be effectively adjusted not only when the LC cell is assembled but also thereafter. The main advantage of this structure is its voltage-controlled tunability, which, at variance with solid-state gratings, permits precise adjustment of optical parameters. As anticipated, the proposed design enables electrically-tuned high peak transmittance (≥95%) for the first diffraction order in the controllable bandwidth covering visible and infrared wavelengths, making optical elements achromatic. Our theoretical approach and conclusions from the numerical simulations are very useful when selecting a liquid crystal *cycloidal* grating for a particular use, as they allow to create a customized specification chart. The latter helps to find suitable parameters to reach the desired operational range of diffraction grating within design in terms of low-cost and compact implementation. Such optimization demonstrates that besides well-known display applications, NLC may be considered as a good material to create efficiently tunable passband optical filters with adjustable bandwidths ideal for use in spectroscopy, optical communication networks, remote sensing systems and beyond. Moreover, the proposed liquid crystalline diffraction gratings may be successively applied as photonic components to control the direction of light beam propagation and operation effectively governed by the electric field. Specifically, taking the advantage of the *cycloidal*-type holographic diffraction grating, such features may be anticipated as selectivity in deflecting light beams for specific diffraction orders, optical signal switching, angular steering and combining moderate response times (in a range of milliseconds) in the systems. Diffraction gratings with assumed periods achievable with the use of available photo-alignment technologies allow for deflection angles up to about 20° for the probe wavelength in the considered spectral range.

## Figures and Tables

**Table 1:** Physical parameters of selected LCs with their values used in numerical simulations.

	6CHBT	E7	5CB (PCB)	MLC-6241-000
K_11_, K_22_, K_33_ [pN]	6.2, 3.7, 10.0	11.1, 5.9, 17.1	6.6, 4.1, 9.7	13.8, 7.5, 21.0 ^1^
ε_‖_, ε_⟂_^2^	12.01, 4.03	19.28, 5.21	17.0, 6.0	Δε = 5.5
*n*_o_, *n*_e_^3^	1.520, 1.671	1.5225, 1.7394	1.5355, 1.7140	1.4756, 1.5614

^1^ K_33_ has been approximated for MLC-6241-000; ^2^ at 1 kHz; ^3^ at the wavelength of 589 nm and at 25 °C.
